# Effect of *Heterodera schachtii* female age on susceptibility to three fungal hyperparasites in the genus *Hyalorbilia*

**DOI:** 10.21307/jofnem-2020-093

**Published:** 2020-09-04

**Authors:** J. Smith Becker, J. Borneman, J. O. Becker

**Affiliations:** 1 Department of Nematology, University of California, 3401 Watkins Drive, Riverside, CA, 92521; 2 Department of Microbiology and Plant Pathology, University of California, 3401 Watkins Drive, Riverside, CA, 92521.

**Keywords:** Biological control, *DoUCR50*, Fungal hyperparasites, *Heterodera schachtii*, *Hyalorbilia* aff. *multiguttulata*, Hyalorbilia spp

## Abstract

Three closely related nematophagous fungi in the genus *Hyalorbilia* were compared for their ability to parasitize females and eggs of *Heterodera schachtii* at different developmental stages. DoUCR50, StM, and ARF were originally isolated from *Heterodera schachtii*, *Meloidogyne incognita,* and *Heterodera glycines*, respectively. Phylogenetic analysis and pairwise sequence analysis showed that DoUCR50 and StM are more closely related to each other than they are to ARF. DoUCR50 parasitism suppressed 100% of the J2 hatch from 3-week-old *H. schachtii* females and 75% of the hatch from 4-week-old females. Eggs within 5-week-old females were resistant to parasitism, and hatch of J2 was unaffected by exposure to DoUCR50. StM and ARF did not reduce the hatch of J2 from *H. schachtii* females of any age. Eggs removed from females and spread onto water agar cultures of the fungi were mostly resistant to parasitism. DoUCR50 parasitized only 16% of such eggs from 3-week-old females. Extracellular hydrolytic enzyme production by the three fungal strains grown on PDA or parasitized *H. schachtii* females was evaluated using API ZYM (bioMérieux) test strips. All three fungi produced extracellular hydrolytic enzymes when grown on PDA or *H. schachtii* females. Trypsin-like protease activity was uniquely detected in DoUCR50 grown on PDA and *H. schachtii* females, with the highest activity associated with the fungus grown on parasitized females.

The ascomycete fungus *Hyalorbilia* aff. *multiguttulata* DoUCR50 (basionym: *Dactylella oviparasitica* DoUCR50) is a hyperparasite of the sugarbeet cyst nematode, *Heterodera schachtii*. It is the causal agent of a long-term population suppression of this nematode in field 9E at the University of California Riverside Agricultural Operations (Borneman and Becker, 2007). *Dactylella oviparasitica* was re-named *Brachyphoris oviparasitica* (Chen et al., 2007) and subsequently assigned to the teleomorphic species *Hyalorbilia oviparasitica* (Baral et al., 2018). DoUCR50 was most recently re-named as *Hyalorbilia* aff. *multiguttulata* (H.-O. Baral, Tübingen, Germany, pers. comm.).

Microscopic studies on the mode of action of DoUCR50 parasitism of *H. schachtii* raised in Arabidopsis or cabbage root tissue culture revealed that sedentary fourth-stage juveniles exposed to the outside of the root are especially susceptible to parasitism (Smith Becker et al., 2013). In contrast, isolated eggs and eggs containing early juvenile stages were less susceptible. In cabbage root culture, DoUCR50 reduced the number of *H. schachtii* females with eggs by more than 95% and the number of eggs per female by almost 60% compared with females developing in culture without the fungus. Similarly, parasitism of *Heterodera glycines* by a sterile nematophagous fungus, ARF-L, was detected at early developmental stages after inoculation of soybean with nematode eggs and juveniles (Timper et al., 1999). Mycelial mats were observed on the surface of sedentary juvenile stages and females. The authors hypothesized that the fungus penetrates the cuticle of the developing nematode directly, similar to the mechanism of infection described for cyst parasitism (Kim et al., 1992). These observations strongly suggest that parasitism of developing late juvenile stages is an important mode of action in the population suppression of cyst nematodes by these fungi.

ARF-L isolate TN-14 (hereafter referred to as ARF) has now also been assigned to the genus *Hyalorbilia* (H.-O. Baral, Tübingen, Germany, pers. comm.). ARF was isolated from 9 out of 95 populations of *H. glycines* in Arkansas in the late 1980s (Kim and Riggs, 1991). Field soil naturally infested with ARF suppressed the number of cysts developing on soybean seedlings by more than 50% compared with seedlings grown in autoclaved soil. In tests with females of *Heterodera* species or egg masses of *Meloidogyne incognita* placed on cultures of the fungus, ARF parasitized eggs of *M. incognita*, *Cactodera betulae*, *H. graminophila*, *H. lespedezae*, *H. leuceilyma*, *H. trifolii*, and *H. schachtii*. An electron microscopic study of the ARF infection process on *H. glycines* females revealed the presence of holes in the cuticle at infection sites, suggesting the involvement of enzymatic degradation. This was the first report of direct penetration by a fungal parasite on a cyst nematode host (Kim et al., 1992). Despite culture on 28 different media and attempts to combine different mating types, no sporulation of ARF strains was observed.

In a subsequent study, 17 isolates of ARF-like fungi collected from *H. glycines* were grouped into two morphological phenotypes, ARF18-C and ARF18-L (Kim et al., 1998). The two phenotypes differed in the appearance of sclerotium-like structures (SLS) produced on CMA. ARF18-C isolates produced compact SLS while ARF18-L produced loosely formed SLS. ARF18-C and ARF18-L also differed in their pathogenicity on soybean cyst nematodes at different stages of development, depending on the type of assay used. In pathogenicity tests using CMA-grown fungi placed on water agar, ARF18-L parasitized approximately half as many eggs in cysts as ARF18-C. In contrast, ARF18-L was more effective at suppressing *H. glycines* nematode numbers and infected more eggs, juveniles, and young females than ARF18-C in assays conducted in the soybean rhizosphere (Timper and Riggs, 1998; Timper et al., 1999). These results highlight the importance of rhizosphere assays for the selection of potential biocontrol strains.

A fungus closely related to DoUCR50 and ARF was suggested to be the causal agent of *M. incognita* suppression in orchards of Lovell peach growing in the San Joaquin Valley of California in 1976. According to the authors, it was the first report of a fungal parasite of root-knot nematode eggs (Ferris et al., 1976; Stirling and Mankau, 1978a). Sporulation in culture was achieved after extensive experimentation with a variety of complex media. Culture on Yeast Peptone Soluble Starch agar enriched with egg yolk and incubation under fluorescent light resulted in good sporulation (Stirling and Mankau, 1978b). The authors identified the novel fungus as *Dactylella* based on conidia morphology, and provided the name *Dactylella oviparasitica*, hereafter referred to as StM. Several distinct strains of similar fungi were obtained which exhibited differences in virulence on *M. incognita*, growth rate, and sporulation. StM preferentially parasitized eggs in clumps or clusters upon removal from the gelatinous matrix of the *M. incognita* female and was less effective at parasitizing single eggs. StM parasitism was not unique to *M. incognita*. The fungus parasitized all *Meloidogyne* species tested, as well as eggs of *Diplenteron* sp., *Acrobeloides* sp., *Tylenchulus semipenetrans*, and *H. schachtii*. StM was also observed to invade white females and cysts of *H. schachtii,* but eggs of *H. schachtii* in older cysts containing J2 were less susceptible to parasitism. Microscopic observation of StM during parasitic interaction with *M. incognita* revealed the presence of appressoria formation on eggs, suggesting a direct penetration mechanism. The ability of the fungus to produce cleared zones on medium containing colloidal chitin raised the possibility that direct penetration of chitin containing eggs was facilitated by chitinase (Stirling and Mankau, 1979). Neither StM, ARF nor our strain DoUCR50 produced trapping structures on dilute Corn Meal Agar (CMA) or water agar, unlike the majority of previously described nematophagous *Dactylella* species (Pramer, 1964).

A phylogenetic analysis of DoUCR50, StM, and ARF revealed that they are members of a clade of similar nematophagous fungi (Yang et al., 2012). This rRNA ITS analysis showed that these three fungi belong to an assemblage of organisms, originally called the DO Clade, with global distribution. In addition to confirming the phylogenetic placement of ARF within the DO Clade, Sharma et al. (2017) created a draft genome assembly of the fungus. Gene prediction analysis was performed, and a total of 14,461 protein-encoding genes were identified. Among the genes identified through BLAST analyses were hydrolytic enzymes, such as serine proteases and chitinases, which have been previously associated with virulence of nematophagous fungi (Yang et al., 2007).

The objective of this study was to compare the parasitism of three genetically similar species from the genus *Hyalorbilia* on *H. schachtii* females and eggs. Previous studies on cyst parasitism have shown that susceptibility to fungal parasitism decreases as females and eggs mature. Our goal was to quantify this effect by testing the susceptibility of females and eggs of uniform age. The role of extracellular hydrolytic enzymes in parasitism was also investigated by testing for the activity of enzymes both in fungi grown on PDA and *H. schachtii* females.

## Materials and methods

### Fungal strains

Three *Hyalorbilia* strains were the focus of the investigations. Strain DoUCR50 originated from the *H. schachtii*-suppressive field 9E at Agricultural Operations, University of California, Riverside, CA (Olatinwo et al., 2006). Strain ARF was initially isolated from parasitized eggs of the soybean cyst nematode, *H. glycines*. It was one of several strains collected in the mid-south region of the United States from 1987 to 1994 (Timper and Riggs, 1998) and was provided by Patricia Timper, USDA-ARS, Tifton, GA. The third strain was first isolated from parasitized root-knot nematode eggs in a *Meloidogyne incognita*-suppressive peach orchard (Stirling and Mankau, 1978a). It was purchased as ATCC 38908 from the American Type Culture Collection (Manassas, VA) and named here StM. During this project, all strains were cultured on PDA or water agar in the dark at 23°C and subcultured monthly. Water agar cultures of the fungi were grown by transferring a 1 cm square block of agar from the growing edge of a PDA culture of the fungus to the center of a 1.5% water agar plate. To help ensure the preservation of parasitism, three-week-old females of *H. schachtii* raised in aseptic root culture were placed on fungal cultures grown on water agar every 6 months. After 10 days, parasitized females were placed individually at the center of PDA plates and were incubated in the dark at 23°C for 30 days prior to subculture.

### Effect of temperature and nutrients on fungal growth

One cm square blocks of agar taken from the growing edge of one-month-old PDA-grown cultures of each fungus were placed in the center of water agar or PDA plates. Plates were placed in incubators at 18, 23, 28, or 33°C. After 10 days, the growth of each culture was measured from the edge of the agar block to the edge of the mycelial growth margin. Three plates of each fungus were used for each temperature tested, and the experiment was repeated twice.

### Phylogenetic and pairwise nucleotide sequence analyses

The sequences used were from *Hyalorbilia* spp. and related groups described in Baral et al. (2018) and from prior studies examining nematophagous fungi (Stirling and Mankau, 1978a; Timper and Riggs, 1998; Yin et al., 2003; Olatinwo et al., 2006). The analyses used the 5.8S rRNA gene and partial 28S rRNA gene sequences. The phylogenetic tree was constructed by performing the following analyses using GenomeNet (https://www.genome.jp/). Sequences were aligned using the CLUSTAL W algorithm (Thompson et al., 1994). The final alignment had a length of 660 nucleotides, and the sequences ranged from 648 to 654 nucleotides. Alignment and phylogenetic reconstructions were performed using the function ‘build’ of ETE3 v3.0.0b32 (Huerta-Cepas et al., 2016) as implemented on the GenomeNet (https://www.genome.jp/tools/ete/). Maximum likelihood trees were inferred using RAxML v8.1.20 that was run with model GTRGAMMA and the default parameters (Stamatakis, 2014). Branch supports were computed out of 100 bootstrapped trees. The tree figure was created using the Interactive Tree of Life (Letunic and Bork, 2007) (https://itol.embl.de/). Pairwise analyses using EMBOSS Water (Smith and Waterman, 1981; Madeira et al., 2019) were performed to determine the % nucleotide sequence identity of rRNA genes and the rRNA ITS region among the three strains. The accession numbers of the sequences used in this analysis are shown in the phylogenetic tree.

### Surface sterilization of *Heterodera schachtii* females

Three-week-old cabbage seedlings grown in 200 cm^3^ sandy loam in cone-tainers (Stuewe & Sons Inc., Tangent, OR) were inoculated with 1,000 freshly hatched J2 of *H. schachtii* per cone and maintained in a greenhouse at 25 ± 2°C with ambient light. At 3, 4, or 5 weeks post-inoculation, roots from two cones were vigorously washed in a 24.5 cm diameter × 5 cm deep stainless-steel pan to dislodge females. The supernatant slurry was poured through a 250 µM mesh sieve (No. 60) to collect females. Females of uniform size were removed from debris using tweezers and placed into 30 ml sterile distilled water overlaid onto a water agar plate containing 100 µg/ml rifampicin and 100 µg/ml ampicillin. Females were stored overnight at 4°C on the antibiotic plates. Forceps were used to individually transfer females to a 70 µM mesh cell strainer (BD Falcon, Becton Dickinson, Franklin Lakes, NJ) placed in 30 ml sterile distilled water in a petri dish. Approximately 200 white females were collected for each experiment. The cell strainer was transferred aseptically to a 100 mm × 15 mm petri plate containing 30 ml of 0.4% sodium hypochlorite. Females were incubated in the dilute bleach solution for 2 min with gentle agitation then transferred in the mesh strainer to a second petri plate containing 30 ml sterile distilled water to rinse. The strainer containing surface-sterilized females was then placed on a water agar plate, and the mesh was cut from the base of the strainer using a flame-sterilized scalpel to facilitate the transfer of the females.

### Effect of female age on J2 hatch after fungal parasitism

Fungal parasitism was evaluated for *H. schachtii* females collected 3, 4, and 5 weeks or approximately 357, 476, and 595 degree-days (DD, 8°C basal temperature (Curi and Zmoray, 1966)) respectively, post-inoculation by measuring J2 hatch inhibition. Surface-sterilized females were distributed to plates containing one-month-old water agar cultures of DoUCR50, ARF, StM, or no fungus as the control. Approximately 30 to 40 females were arranged evenly spaced around the center of the plate, 0.5 to 1 cm from the edge of the fungal block, and plates were incubated in the dark at 23°C. Healthy surface-sterilized females were placed on water agar plates as controls. After 3 days, 25 parasitized or control females from each plate were transferred to the center of water agar plates, 5 females per plate, and 5 plates per fungal strain or control. Plates were incubated in the dark at 23°C for 6 days, after which females were removed from plates and used for enzyme assays. Hatched J2 were rinsed from plates with 3 × 5 ml water and counted. The experiment was repeated twice.

### Effect of female age on parasitism of isolated *H. schachtii* eggs

Approximately 100 surface-sterilized females collected 3, 4, or 5 weeks post-inoculation were placed into a sterile 70 µM mesh cell strainer (Falcon) on a water agar antibiotic plate (100 µg/ml rifampicin, 100 µg/ml ampicillin) overlaid with 20 ml sterile distilled water. Females were ruptured on the sieve using sharp flame-sterilized tweezers. The sieve was agitated to allow eggs to pass through the mesh onto the agar. Eggs were rinsed five times with sterile distilled water to remove debris. For each rinse, 30 ml sterile water was added to the plate and eggs were gently agitated. Water and debris were removed using a 23 cm sterile glass Pasteur pipette. After the final rinse, eggs were resuspended in 10 ml sterile water and transferred to a 50 ml sterile disposable centrifuge tube. The volume of water in the tube was adjusted to provide 1 egg/µl water. Each of the fungal strains (DoUCR50, ARF, and StM) was grown on water agar at 23°C for 20 days, after which the mycelia covered about half the plate or more. Ten µl of egg suspension was spotted onto five sites on each plate, approximately 2 cm from the center of the plate. Each of these sites served as replicates for the experiment. A fine pointed dental tool was used to move eggs such that each spot contained 10 eggs, and they were arranged such that they were not touching each other. Plates were incubated for 10 days at 23°C in the dark, and the eggs were monitored for parasitism and hatching of J2. The experiment was repeated twice.

### Production of hydrolytic enzymes by fungi grown on PDA or *H. schachtii* females

The API ZYM test kit (bioMérieux, France) provides a semi-quantitative assay for the presence of 19 hydrolytic enzymes: alkaline phosphatase, esterase, esterase lipase, lipase, leucine arylamidase, valine arylamidase, cysteine arylamidase, trypsin, chymotrypsin, acid phosphatase, naphthol-AS-BI-phosphohydrolase, α-galactosidase, b-galactosidase, α-glucosidase, β-glucosidase, N-acetyl-β-glucosaminidase, α-mannosidase, and α-fucosidase. DoUCR50, ARF, and StM strains grown either on PDA or 3-week-old *H. schachtii* females were tested for extracellular hydrolytic enzyme production as follows. Sixty-five µl of 0.85% NaCl suspension medium was added to each well of the API ZYM test strips. For assay of enzymes produced by PDA-grown fungi, 5 mm cubes of PDA containing mycelia from 1-month-old cultures were placed into the test wells, one cube per well. Cubes of PDA were used as control. Enzyme production by fungi grown for 9 days on 3-week-old *H. schachtii* females was assayed by placing a single parasitized female or healthy control female from the hatch inhibition assay into each well of the test strips. Test strips were incubated at 37°C in the dark for 4 hr. The reaction was stopped by the addition of one drop of ZYM A reagent followed by one drop of ZYM B. Color development was allowed to proceed for 10 min at 23°C before recording. The activity of each enzyme was recorded on a colorimetric scale provided by the manufacturer ranging from 0 (no activity) to 5 (maximum), and the experiment was repeated three times.

### Statistical analysis

Data sets of repeated experiments were analyzed for homogeneity of variance with Levene’s test (Real Statistics Using Excel: © 2012-2019, Charles Zaiontz, http://www.real-statistics.com). As there were no statistical differences between the variances of the repeated experiments (*P* ≤ 0.05), the homogeneity of variance assumption was met. Treatment effects were analyzed using a *t*-test or one-way analysis of variance (ANOVA) procedure. If appropriate, means were compared using Fisher’s protected least significant difference (LSD) test (*P* ≤ 0.05). Standard error bars with the same lowercase letter indicate no significant difference. Enzyme color rating means were analyzed by the nonparametric Kruskal–Wallis test, followed by Dunn’s post hoc test to determine the differences among the groups (*P* < 0.05).

## Results

### Effect of temperature and nutrients on fungal growth

**Figure 1:**
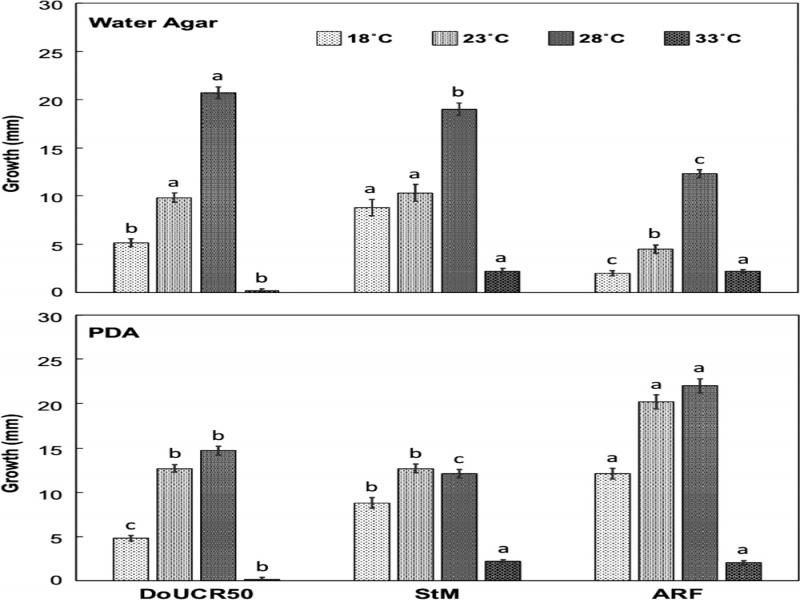
Effect of temperature and nutrients on rate of growth of *Hyalorbilia* strains DoUCR50, StM, and ARF in agar culture. Mycelial growth from blocks of PDA cultures placed on PDA or water agar and grown at 18, 23, 28, or 33°C was measured after 10 days. The experiment was repeated twice, and data were pooled. Standard error bars are shown.

All three fungal strains grew fastest at 28°C on both water agar and PDA, and growth was strongly attenuated at 33°C (Fig. 1). The three strains differed in their rate of growth on water agar and PDA. Both DoUCR50 and StM grew faster on water agar than on PDA at 28°C, while the growth of ARF on water agar at 28°C was approximately 50% that of DoUCR50 and StM. On PDA, the rate of growth of ARF at 23°C or 28°C was almost twice that of DoUCR50 or StM.

### Phylogenetic and pairwise nucleotide sequence analyses

**Figure 2:**
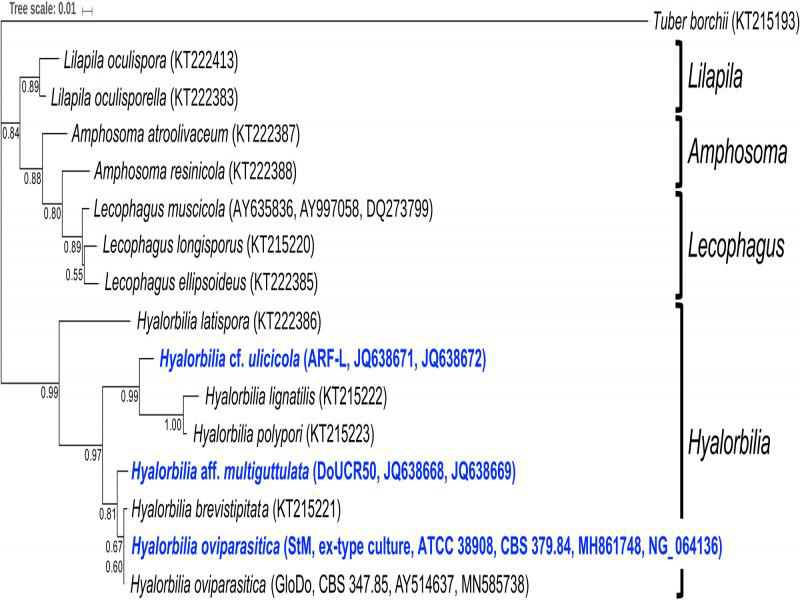
Maximum likelihood tree depicting the phylogenetic relationships of three nematophagous fungi and related members of the *Orbiliomycetes*. The outgroup is *Tuber borchii*. Bootstrap values (out of 100) are shown on the branches. Branch lengths are the expected number of substitutions per nucleotide.

A phylogenetic analysis of rRNA genes and the rRNA ITS region showed that all three fungi belong to an assemblage of fungi in the genus *Hyalorbilia,* which is a member of the class *Orbiliomycetes* (Fig. 2). This group also includes a closely related fungus (*H. oviparasitica*, GloDo, CBS 347.85) isolated from *Globodera rostochiensis* in the Netherlands (Chen et al., 2007), suggesting that it might be nematophagous. This phylogenetic analysis and a pairwise sequence analysis (Table 1) both showed that DoUCR50 and StM are more closely related to each other than they are to ARF.

**Table 1.** Percent nucleotide sequence identities of *Hyalorbilia* strains DoUCR50, StM, and ARF.

	DoUCR50 (%)	StM (%)	ARF (%)
DoUCR50	100	**98.0**	**93.9**
StM	*75.5*	100	**93.4**
ARF	*72.6*	*69.5*	100

**Note:** Analyses of partial 28S rRNA genes are shown in bold and analyses of the rRNA ITS region are shown in italic.

### Effect of female age on J2 hatch after fungal parasitism

**Figure 3:**
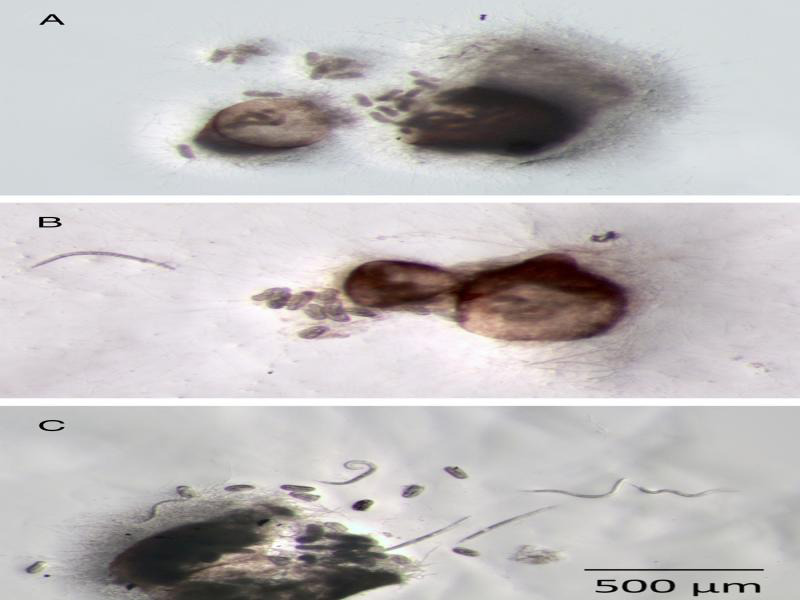
Growth of *Hyalorbilia* strains on 3-week-old *Heterodera schachtii* females. Three-week-old *H. schachtii* females were placed on water agar cultures of DoUCR50 (A), StM (B), or ARF (C). After 3 days, females were transferred to water agar and incubated for an additional 6 days at 23°C. Parasitized females were cut in half with a scalpel blade and incubated for 24 hr at 23°C prior to being photographed.

**Figure 4:**
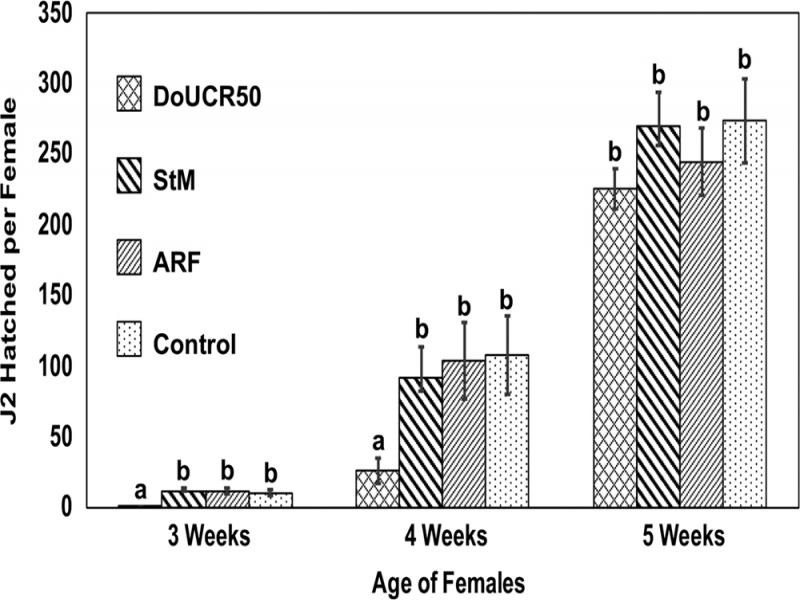
Effect of *Heterodera schachtii* female age on susceptibility to J2 hatch suppression by *Hyalorbilia* strains DoUCR50, StM, and ARF. For each treatment, 25 *H. schachtii* females were placed on water agar (Control) or water agar cultures of fungi for 3 days at 23°C. Females were transferred to water agar plates, five females per plate, for an additional 6 days to monitor J2 hatch. The experiment was repeated twice, and the data were pooled. For each time point, treatments with the same letter are not significantly different (*P* < 0.05).

All three fungal strains colonized 3, 4, and 5-week-old *H. schachtii* females placed on water agar cultures of the fungi. Fungal hyphae grew densely on the surface of females and within the gelatinous matrix inside of females (Fig. 3). Parasitized eggs were occasionally observed in ARF and StM colonized females, but most eggs appeared healthy after exposure to these two fungal strains regardless of the age of the females. In contrast, DoUCR50 parasitized all of the eggs in 3-week-old females and was observed to parasitize some eggs within 4 and 5-week-old females. Many eggs within DoUCR50-colonized females were engulfed in a thick mass of mycelia, preventing enumeration of parasitized eggs. Only DoUCR50 caused significant suppression of hatch from parasitized females (Fig. 4). Eggs of 3-week-old females were most susceptible, with complete inhibition of J2 hatch, and resistance to parasitism increased as the females matured. The hatch of J2 from 4-week-old females parasitized by DoUCR50 was reduced by 75% while hatch of J2 from 5-week-old females was not significantly different from that of control females. Neither ARF nor StM caused a reduction of J2 hatch in females of any age compared to no fungus control females. The average number of J2 hatching per control female at 3, 4, or 5 weeks of age during the six-day observation period was 11, 108, and 274, respectively.

### Parasitism of isolated eggs

**Figure 5:**
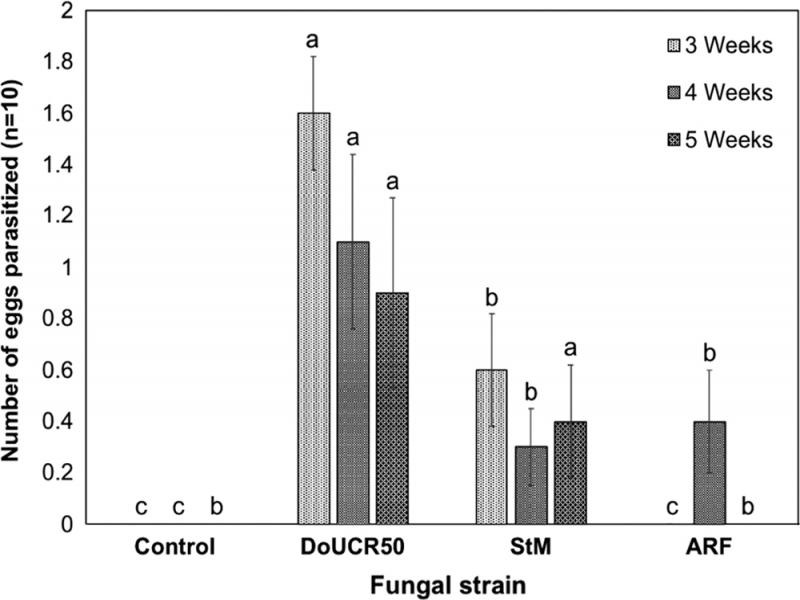
Effect of *Heterodera schachtii* female age on susceptibility of isolated eggs to parasitism by DoUCR50, StM, or ARF. Five groups of 10 eggs from females collected 3, 4, or 5 weeks after inoculation of cabbage were spotted onto water agar cultures of the fungi or on water agar without fungus (Control). Plates were incubated for 10 days at 23°C in the dark, and the eggs were monitored for parasitism and hatching of J2. Data are reported as the average number of parasitized eggs per group of 10. The experiment was repeated twice, and the data were pooled. Standard error bars with the same lowercase letter indicate no significant difference among fungi of a respective week.

Eggs removed from females and placed on water agar cultures of the fungi were mostly resistant to parasitism (Fig. 5). The highest rate of egg parasitism, 16%, occurred with eggs from 3-week-old females placed on DoUCR50 although differences in parasitism of isolated eggs were not statistically significant among treatments.

### Assay for hydrolytic enzymes

One parasitized female or one 5 mm cube of PDA-grown fungus per sample well produced sufficient extracellular enzyme activity to compare treatments in API ZYM test strips. Only the assay for naphthol-AS-BI-phosphohydrolase produced a positive reaction with control PDA agar (data not shown). This was possibly an artifact, as enzyme activity is unlikely to be preserved after autoclaving. The colorimetric value for this enzyme was also consistently high in all treatments. Uninfected female *H. schachtii* produced only low levels of activity (values less than 2) for acid phosphatase, α-glucosidase, and β-glucosidase (Table 2). All three fungal strains grown on PDA or *H. schachtii* females produced activity that was not significantly different among the treatments for alkaline phosphatase, L-arylamidase, V-arylamidase, acid phosphatase, and naphthol-AS-BI-phosphohydrolase (data not shown). In general, the activity of α-glucosidase was higher in PDA-grown fungi while the activity of β-glucosidase was greater in fungi grown on *H. schachtii* females, although the differences were not all statistically significant. Colorimetric values for n-acetyl-β-glucosaminidase, a proxy for chitinase activity, were higher for samples containing fungi grown on females than fungi on PDA cubes. The only enzyme activity that was qualitatively different among treatments was trypsin. Trypsin activity was detected in treatments containing DoUCR50 grown on either PDA or *H schachtii* females but not in any other treatment. The colorimetric value for trypsin activity produced by DoUCR50 growing on *H. schachtii* females was higher than that of the fungus growing on PDA.

**Table 2.** API ZYM assay for extracellular hydrolytic enzyme activity.

Treatment^a^	Trypsin	Acid phosphatase	α-glucosidase	β-glucosidase	N-acetyl-β-glucosamidase
C PDA	0.0 ± 0.00 c	0.0 ± 0.00 b	0.0 ± 0.00 c	0.0 ± 0.00 c	0.0 ± 0.00 c
C Fem	0.0 ± 0.00 c	1.3 ± 0.33 ab	0.7 ± 0.67 bc	0.3 ± 0.33 bc	0.0 ± 0.00 c
DoUCR50 PDA	2.3 ± 0.33 b	3.7 ± 1.33 a	3.7 ± 0.33 a	1.7 ± 0.88 abc	1.3 ± 0.33 b
DoUCR50 Fem	3.7 ± 0.33 a	3.0 ± 0.00 ab	2.3 ± 0.67 ab	3.3 ± 0.33 a	3.3 ± 0.33 a
ARF PDA	0.0 ± 0.00 c	3.0 ± 0.00 ab	3.3 ± 0.33 a	1.3 ± 0.33 abc	1.7 ± 0.33 b
ARF Fem	0.0 ± 0.00 c	3.4 ± 0.33 ab	2.0 ± 0.58 abc	3.0 ± 0.00 ab	3.3 ± 0.33 a
StM PDA	0.0 ± 0.00 c	3.7 ± 1.33 a	3.3 ± 0.67 a	2.3 ± 0.33 ab	2.0 ± 0.58 ab
StM Fem	0.0 ± 0.00 c	3.4 ± 0.33 ab	1.0 ± 0.00 abc	2.7 ± 0.88 ab	3.3 ± 0.33 a

**Notes:** *Hyalorbilia* strains (DoUCR50, StM, ARF) were grown on PDA or *Heterodera schachtii* females (Fem) prior to assay for enzyme activity in API ZYM test strips. Enzyme activity was scored on a colorimetric scale from 1 to 5 using a chart provided by the manufacturer. Enzymatic tests were carried out in triplicate and means were analyzed by the nonparametric Kruskal–Wallis test, followed by Dunn’s post hoc test to determine the differences among the groups (*P* < 0.05). Means (± standard error) followed by different letters within a column represent significant differences. ^a^C PDA: control PDA cube (no fungus); C Fem: control *H. schachtii* female (no fungus); DoUCR50 PDA: strain DoUCR50 on PDA cube; DoUCR50 Fem: *H. schachtii* female parasitized by strain DoUCR50; ARF PDA: Strain ARF on PDA cube; ARF Fem: *H. schachtii* female parasitized by strain ARF; StM PDA: Strain StM on PDA cube; StM Fem: *H. schachtii* female parasitized by strain StM.

## Discussion

The three *Hyalorbilia* strains, DoUCR50, ARF, and StM, are closely related nematophagous fungi originally isolated from agricultural field locations with soils suppressive to different sedentary endoparasitic nematodes. Although sporulation of StM was observed when initially isolated, the culture obtained from ATCC never sporulated, even when grown on the appropriate media. Attempts to induce sporulation of DoUCR50 and ARF have also been unsuccessful, despite exhaustive efforts on a variety of media and culture conditions. A phylogenetic analysis of the three strains demonstrated that they all belong to the genus *Hyalorbilia*. This analysis, along with a pairwise sequence analysis, revealed that DoUCR50 and StM are more closely related to each other than they are to ARF. Comparison of growth characteristics of the fungi also showed that DoUCR50 and StM were more similar to each other than they were to ARF, which grew faster on PDA than the two California strains but did not grow as well on water agar.

The three fungal strains colonized the females of *H. schachtii* and grew within the gelatinous matrix inside of the females, but only DoUCR50 caused a reduction in hatch of J2. Fungal growth was observed on some eggs within females colonized by StM and ARF, but these were most likely eggs that were nonviable since the hatch of J2 from colonized females was the same as from uninfected controls. Both StM and a fungus similar to ARF, ARF18, had previously been reported to parasitize eggs within *H. schachtii* females, but the effect on J2 hatch was not evaluated (Stirling and Mankau, 1979; Kim and Riggs, 1991). Although it is possible that StM and ARF lost virulence during long-term storage, an alternative explanation for the lack of virulence in our assays may be differences in parasitism tests used here and in previous studies. We used fungi growing on water agar to simulate the low nutrient environment of the soil. Trap forming nematophagous fungi in the family *Orbiliaceae* typically produce traps predominately after transfer to dilute nutrient agar, suggesting that the signal to transition from saprophytic growth to parasitic growth is in part triggered by low nutrient conditions (Niu and Zhang, 2011). This effect was first reported in the study of the nematophagous fungus *Arthrobotrys oligospora* (Woronin, 1870; Zopf, 1888). Previous parasitism tests of StM and ARF on *H. schachtii* relied on the placement of cysts directly on fungi grown on full or half-strength PDA or CMA for assessment of parasitism (Stirling and Mankau, 1979; Kim et al., 1998). ARF grown on PDA was significantly more virulent to eggs within cysts of *H. glycines* than when grown on CMA, demonstrating that fungal parasitic potential was influenced by nutritional status.

The modulation of fungal virulence by nutritional status or other signals may help to explain the differential susceptibility of nematode eggs to DoUCR50 depending on whether they are contained within the female or isolated and spread out on a water agar culture of the fungus. Mycelia of DoUCR50 grew densely on the surface and inside of females, presumably using the gelatinous matrix as a nutrient source. In 3-week-old females, the susceptibility of eggs to parasitism was much greater when confined within the female. Isolated eggs of the same age spread over the surface of water agar cultures of DoUCR50 were mostly resistant to parasitism. We hypothesize that this is due to the induction of virulence factors in the fungus during colonization of the female. If fungal virulence is dependent upon the induction of specific factors during colonization of the female, the isolated eggs may escape parasitism because they do not elicit the necessary virulence factors. Potential candidates for virulence factors in nematophagous fungi include toxins (Degenkolb and Vilcinskas, 2016) and hydrolytic enzymes such as chitinase and protease (Yang et al., 2007).

The API ZYM test kit provided a semi-quantitative assay for hydrolytic enzyme production by the three fungi growing on PDA or while parasitizing *H. schachtii* females. One limitation of our study was that we did not have a way to standardize the fungal content in PDA cubes vs. mycelia growing in parasitized females. However, the quantity of mycelia in a single three-week-old female, approximately 0.5 cubic mm, was most likely less than the amount in a 5-mm cube of PDA-grown culture. Thus, we presume that higher enzyme activity levels in samples containing parasitized females represent an induction of activity over that of the fungus growing on PDA. All three fungi produced N-acetyl-β-glucosaminidase, indicative of chitinase activity. The relative level of activity was higher in fungi growing on females, suggesting that the enzyme may be induced during the infection process. Chitin is a structural component of nematode eggs, and chitinase was found to be a virulence factor in other nematophagous fungi (Yang et al., 2007). Interestingly, only DoUCR50 produced measurable levels of trypsin-like protease activity, and the activity was relatively higher in the fungus growing on *H. schachtii* females. Although StM and ARF lacked measurable trypsin activity, it is too early to speculate on the correlation between the absence of trypsin activity and the reduced parasitism of these two strains on *H. schachtii*. Both fungi were initially isolated from other nematode hosts, and their lack of virulence on *H. schachtii* may be due to as yet uncharacterized host-specific factors.

The first serine protease isolated from a nematophagous fungus was purified from *Metapochonia suchlasporia* (syn. *Verticillium suchlasporium*) (Lopez-Llorca, 1990). The protease was immunolocalized to hyphae and appressoria that were penetrating eggshells during infection of *H. schachtii* eggs, providing evidence that the enzyme was involved in parasitism (Lopez-Llorca and Robertson, 1992). Similarly, a subtilisin-like serine protease, VCP1, was produced by the fungus *Pochonia chlamydosporia* during the early infection process on *M. incognita* eggs (Segers et al., 1996; Ward et al., 2012). The purified protease removed the outer proteinaceous vitelline membrane of *M. incognita* eggs, rendering them susceptible to parasitism. Experiments in our labs are currently underway to evaluate the expression of fungal proteases and other genes during the parasitism process using RNAseq. Identification of genes upregulated during this process will help in understanding the relative importance of hydrolytic enzymes and other virulence factors during fungal parasitism of the nematode.

One objective of this study was to quantify the effect of *H. schachtii* female age on the susceptibility of eggs to parasitism. Timper et al. (1999) showed that up to 72% of *H. glycines* J4 and pre-gravid females were parasitized by ARF in the soybean rhizosphere during the first 2 weeks of nematode development. This early rate of parasitism was comparable to the rate observed on white females during the third week after inoculation. Chen and Chen (2003) tested the susceptibility of *H. glycines* females and cysts to more than 10 species of parasitic fungi, including ARF. They found that younger eggs containing embryos were more vulnerable to fungal parasitism than eggs containing J1 or J2. Increased resistance to parasitism with developmental age had previously been noted for StM and ARF, but the age of females and cysts was estimated based on appearance (Stirling and Mankau, 1979; Kim and Riggs, 1991). Although white females are typically younger than brown cysts, the color of females and cysts is not always indicative of the developmental stage of eggs within (Tribe, 1977). In this study, synchronously inoculated cabbage roots provided a source of females of defined age for determination of egg susceptibility to the fungi. Eggs within females obtained from roots 3 weeks after inoculation (approximately 357 DD) were all susceptible to DoUCR50 parasitism, and the hatch was completely suppressed. By 5 weeks after inoculation (about 595 DD), eggs within females were completely resistant to parasitism, and J2 hatch was unaffected by DoUCR50. Browning of the female cuticle is thought to provide a protective barrier for developing eggs, yet all females collected 5 weeks post-inoculation on cabbage roots were still white. The developmental stage of eggs may be a more important determinant of resistance to fungal parasitism than the barrier provided by the browning of the female cuticle wall. The primary benefit of the browning reaction may be resistance to desiccation (Clarke, 1970).

The resistance of eggs containing J1 or J2 to parasitism may be due to increasing impermeability of the eggshell during development rather than the resistance of the juvenile to parasitism, as the composition of the juvenile cuticles is chemically similar to that of the female and cyst walls (Clarke, 1970). Nematode eggshells are comprised of an inner lipid layer, a middle chitin containing layer, and an outer proteinaceous vitelline layer (Wharton, 1980; Bird and Bird, 1991). The inner lipid layer provides the most significant barrier to permeability, while the chitin layer provides structure (Bird and McClure, 1976; Wharton, 1980). In *H. glycines*, eggshell development is an ongoing process that continues through embryogenesis (Burgwyn et al., 2003). Mature eggs are much less permeable than early embryonic eggs, suggestive of changes in structure during development. We hypothesize that the permeability of the eggshells of undifferentiated and recently fertilized eggs may render them more susceptible to fungal attack. Likewise, the vulnerability of sedentary J4 to parasitism may be attributed to the loss of the protective barrier afforded by the mature eggshell.

In summary, three closely related nematophagous fungi show differing potential as parasites of *H. schachtii*. Only DoUCR50, originally isolated from *H. schachtii*, was able to reduce the hatch of J2 in our in vitro tests. DoUCR50 was unique among the three fungi in the production of trypsin-like protease activity. Complete genome sequence analysis of DoUCR50, ARF, and StM is currently in progress and should help to provide additional information on genes associated with parasitism in the three strains.
